# Controlling caspase activity in life and death

**DOI:** 10.1371/journal.pgen.1006545

**Published:** 2017-02-16

**Authors:** Kristin White, Eli Arama, J. Marie Hardwick

**Affiliations:** 1 Cutaneous Biology Research Center, Massachusetts General Hospital Research Institute/ Harvard Medical School, Boston, Massachusetts, United Sates of America; 2 Department of Molecular Genetics, Weizmann Institute of Science, Rehovot, Israel; 3 Johns Hopkins University Bloomberg School of Public Health, Baltimore, Maryland, United States of America; The University of North Carolina at Chapel Hill, UNITED STATES

Every day, billions of human cells terminate their normal activities and launch intrinsic suicide pathways. Timely cell death is orchestrated by destructive functions encoded by dying cells, such as caspase proteases in the case of apoptotic or pyroptotic cell death. Caspases cleave specific intracellular substrates to kill and dismantle cells destined for elimination, and their dysregulation leads to a range of human disorders [1]. *Drosophila* models have proven to be key tools for understanding the regulation of caspases in cell death, but have also revealed other unanticipated roles for caspases, including cell proliferation [2], sperm maturation [3] and neuronal pruning [4]. Furthermore, the presence of widespread, nonlethal caspase activity in fly tissues suggests that there could be additional caspase-dependent processes besides those that are already known [5]. Thus, a new challenge is to understand the connections between non-death and pro-death functions of caspases. A new study from the Bergmann lab [6] reveals that mono-ubiquitylation of the *Drosophila* caspase Dronc inhibits apoptosis; more surprisingly, mono-ubiquitylation also inhibits an alternative role of Dronc. This work uncovered a role for Dronc in several non-lethal activities and organismal survival that apparently does not require Dronc’s protease activity.

## Presumed regulation of cell death by caspase degradation

Three of the seven *Drosophila* caspases (Dronc, Dredd, and Strica) appear to be similar to mammalian “initiator” caspases that cleave and activate “effector” caspases causing apoptosis [7], whereas the remaining four *Drosophila* caspases resemble effector caspases. The main initiator caspase Dronc (homologous to mammalian caspases-2 and -9) plays a major role in activating cell death during fly development and following injury by activating the effector caspases Drice and Dcp-1 (homologous to mammalian caspases-3 and -7) [8]. Dronc activation is dependent on direct binding of its N-terminal caspase recruitment domain (CARD) to the CARD domain in the adapter protein Dark (homologous to mammalian Apaf1), together forming the death-inducing apoptosome [9].

Caspases are tightly controlled by several negative regulatory mechanisms. Active Dronc, presumably derived from the apoptosome, is inhibited by ubiquitylation, a process largely dependent on the E3 ubiquitin ligase Diap1 (*Drosophila* inhibitor of apoptosis; homologous to human XIAP, cIAP1/2). The proposed mechanism is the poly-ubiquitylation of Dronc by Diap1 to trigger either Dronc degradation via the proteasome or its inhibition by other means [10, 11]. However, there is a lack of clarity in the mechanisms of Dronc regulation, as both the proteasome and autophagy pathways must be ablated to stabilize Dronc levels [12].

## Non-degradative inhibition of Dronc by mono-ubiquitylation

Prior to apoptosome formation, unprocessed Dronc is negatively regulated by ubiquitylation; surprisingly, this does not require Dronc degradation [11]. Research from the Bergmann lab [6] sheds light on the regulation of Dronc activity by mono-ubiquitylation, rather than poly-ubiquitylation, in living cells. Mono-ubiquitylation of mammalian caspases has been described [13], and is also thought to alter function rather than promote degradation [14].

Kamber Kaya et al. [6] report that overexpressed Dronc in living tissues is mono-ubiquitylated on lysine 78 (K78) in the CARD domain. Using mass spectrometry and immunoblot analysis, they detected mono- but not poly-ubiquitylated Dronc in developing larval and pupal extracts, a finding further supported by in vitro ubiquitylation on K78 in extracts. Based on a recent crystal structure analysis, K78 forms an intermolecular hydrogen bond with the Dronc residue that directly contacts Dark at the center of the CARD–CARD interface between Dronc and Dark [9]. Therefore, mono-ubiquitylation at lysine 78 is likely to alter or inhibit apoptosome formation and subsequent cell death. Consistent with mono-ubiquitylation being exclusive to living cells, Kamber Kaya et al. [6] failed to find any lysine 78–ubiquitylated Dronc in larval and pupal extracts after *hs-hid* induced apoptosis. Although mutation of the lysine 78 site resulted in a strong inhibition of mono-ubiquitylation, residual mono-ubiquitylation suggested that other sites might be modified when the major site is mutated.

This work [6] provides considerable additional genetic and biochemical data indicating that Dronc K78 mono-ubiquitylation inhibits apoptosis. Overexpression of Dronc and Dark in the eye-antenna imaginal disc epithelial tissues triggers massive cell death in the developing eye, providing a model system to dissect function [10]. Ubiquitylation-resistant Dronc (K78R) was demonstrated to enhance cell death in the developing eye and pupal lethality more than wild-type (WT) Dronc expressed at similar levels. The enhanced apoptotic function of Dronc K78R correlates with higher proteolytic function in vivo, but this is not due to higher inherent catalytic activity, as recombinant proteins cleave Drice similarly in vitro [6]. The possibility that mono-ubiquitylation inhibits Dronc—Dark interaction is suggested by increased binding of K78R to Dark in co-immunoprecipitations [6], consistent with structural data [9].

**Fig 1:**
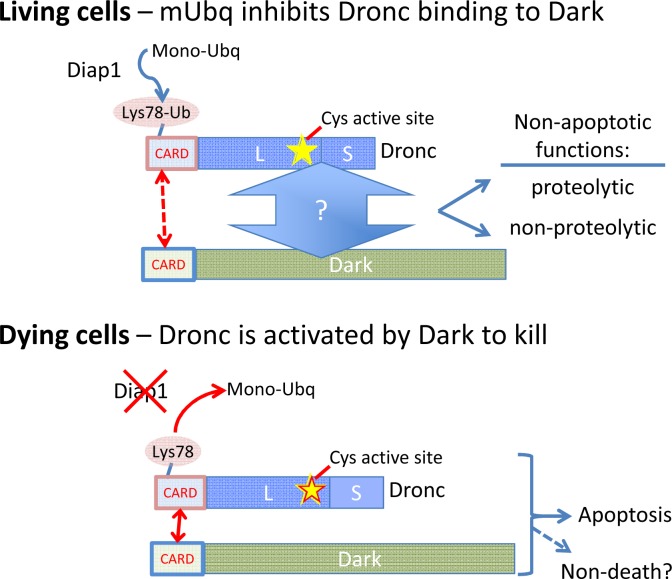
The caspase Dronc functions in both living and dying cells. The *Drosophila* caspase Dronc, which is required for fly development, is composed of an N-terminal CARD domain, a large subunit (L) containing the Cys active site required for proteolytic catalysis, and a small subunit (S) that is auto-catalytically cleaved from the precursor zymogen following recruitment to the Dark apoptosome. The ubiquitin ligase Diap1 mono-ubiquitylates lysine 78 in the CARD domain, which inhibits cell death by interfering with formation of the Dronc–Dark apoptosome. In contrast to non-apoptotic functions of Dronc in some model systems, Dronc’s proteolytic activity is unexpectedly not required for Dronc’s role in apoptosis-induced cell proliferation. Surprisingly, mono-ubiquitylation also suppresses this non-apoptotic function of Dronc, as Dark is still required. Thus, an alternative CARD-independent interaction with Dark or other factor could potentially mediate the observed non-death functions of Dronc.

The E3 ubiquitin ligase responsible for mono-ubiquitylation of Dronc K78 may be Diap1. This is supported by the finding that heterozygosity for *diap1* reduced Dronc mono-ubiquitylation [6]. Additionally, Diap1 is sufficient to mono-ubiquitylate Dronc in vitro. Thus, it appears that in living cells, Diap1 can inhibit apoptosome formation through mono-ubiquitylation of Dronc. However, Dronc inhibition by mono-ubiquitylation has additional functions besides inhibiting apoptosis (Fig 1).

## Non-apoptotic, non-catalytic caspase activity

The authors of this study [6] used several strategies to investigate a physiological role for Dronc mono-ubiquitylation. Ubiquitous expressions of WT and mutant forms of Dronc were used to rescue organismal lethality of *dronc* null flies. Loss of zygotic *dronc* is known to cause a semi-lethal phenotype, with most animals dying during pupal stages, late in development [8]. Although these animals have extra cells, it is unknown whether the lack of cell death is the cause of organismal lethality. However, it was expected that organismal survival required the elimination of some cells by caspases because mutation of the catalytic cysteine (C318A), which abolishes enzymatic activity, also abolished rescue. Unexpectedly, Kamber Kaya et al. [6] found that catalytically inactive Dronc that cannot be mono-ubiquitylated (K78R/C318A) rescued the lethality of *dronc* nulls—a finding that challenges the general assumption that Dronc’s proteolytic activity is essential for normal organismal development. Rescue of lethality by Dronc K78R/C318A is not due to a restoration of catalytic function or of increased apoptosis-inducing activity as extra cells remain, at least in the developing eye and the developing wing [6]. This suggests that mono-ubiquitylation also inhibits non-apoptotic functions of Dronc that are essential for the survival of the organism. Further experimentation using knock-in mutations will be required to confirm this hypothesis.

### Compensatory cell proliferation

To test for non-apoptotic functions of Dronc, Kamber Kaya et al. [6] investigated a phenomenon known as apoptosis-induced compensatory cell proliferation (AiP), which occurs when apoptotic cells release signals that promote proliferation of surrounding living cells. This process was discovered in imaginal discs manipulated to activate Dronc while suppressing effector caspases [2], revealing a non-apoptotic role for Dronc in triggering the proliferation of neighboring cells. The mechanisms are still under investigation, but are suggested to involve autocrine/paracrine Dronc-dependent activation of the c-Jun N-terminal kinase (JNK) pathway, which in turn activates conserved signaling pathways, possibly requiring secretion of tumor necrosis factor (TNF) ortholog Eiger by local activated macrophages (hemocytes) [15]. AiP-related processes have also been reported in mammals and are suggested to contribute to tumorigenesis [16].

Although the catalytically dead C318A mutant of Dronc fails to cause head overgrowth via AiP, blocking mono-ubiquitylation of catalytically inactive Dronc (K78R/C318A) rescues AiP [6]. The proliferation-promoting activity of Dronc K78R/C318A is Dark dependent [6]. Thus, some non-apoptotic functions of Dronc may not require caspase activity when the interaction between Dronc and Dark is strengthened through the inhibition of mono-ubiquitylation. This suggests that the Dark–Dronc complex may recruit other proteins that are important for non-apoptotic caspase functions. In addition, other caspases may compensate for the lack of Dronc in some essential proteolytic functions, and be activated by the mutant Dronc. For example, Strica is known to function with Dronc during certain developmental events [17] and in some types of cell competition [18], which could be tested by removing both Dronc and Strica.

Consistent with this new study from Bergmann and colleagues, there are reports of catalytically independent non-apoptotic functions of fly and mammalian caspases [19, 20]. Catalytically inactive Dronc was shown to inhibit the mitogenic potential of the conserved Numb protein in *Drosophila* neural stem cells [19]. In this paradigm, enforced expression of a phospho-mimetic form of Numb attenuated endogenous Numb activity and caused ectopic neuroblast formation, which was suppressed by overexpression of either WT or catalytically inactive forms of Dronc.

### Sperm development

Although the catalytic activity of Dronc was not required to rescue pupal lethality and AiP if mono-ubiquitylation was blocked, Dronc K78R/C318A failed to rescue male fertility defects in *dronc* null flies [6]. Thus, Dronc catalytic activity may be required for some non-apoptotic roles of Dronc, such as the non-apoptotic caspase function required for spermatid terminal differentiation in *Drosophila* called individualization [3]. In this process, the syncytial spermatids shed much of their cytoplasm and unneeded organelles into a sack-like structure dubbed the “waste bag,” which is conceptually reminiscent of an apoptotic corpse [3]. In contrast to AiP, this extrusion of cytoplasmic contents requires effector caspases in addition to the apoptosome, plus the activity of Cullin-3 [21, 22]. However, a role for mono-ubiquitylation in regulating spermatid individualization is not yet known.

## Future directions

Bergmann and colleagues’ study [6] opens up new directions for research in the regulation of non-apoptotic caspase functions. In addition to the model systems tested in their study, other non-death functions of caspases in flies and mammals will require further examination to distinguish between proteolytic and non-proteolytic caspase functions. For example, the notion that the *Drosophila* apoptotic system might be involved in determining the number of external sensory bristles (macrochaetae) stems from observations of extra macrochaetae in flies mutant for *dark*, *dronc* and cytochrome *c*, but growing evidence implicates non-apoptotic functions of these factors involving the regulation of cell signaling pathways (reviewed in [23]). Restricted activation of caspases is suggested to prune dendritic spines on neurons in the fly without killing the neuron [4]. However, caspases have also been implicated prior to the pruning steps [24], and the requirements for proteolytic activity can be difficult to evaluate, as demonstrated in the new study by Bergmann et al. [6]. Mammalian caspases and regulators of caspase activation have been demonstrated to regulate neuronal activity independent of apoptosis [25, 26], but whether these caspase functions are limited to their proteolytic activities has not been thoroughly investigated.

The most striking finding of the new work by Bergmann and colleagues [6] is that Dronc’s essential function during development may not be its proteolytic apoptosis function. However, this interpretation could be confounded by the presence of residual maternally-loaded Dronc protein in the zygotic *dronc* nulls [8]. Although zygotic *dronc* nulls are semilethal at pupal stages, mutants lacking both maternally loaded and zygotic Dronc are lethal earlier during embryonic stages, and lack almost all embryonic apoptosis. Kamber Kaya et al. do not report that the Dronc K78R/C318A mutant rescued this maternal/zygotic *dronc* null phenotype. In theory, overexpression of the Dronc K78R/C318A mutant could stabilize or activate maternally loaded WT Dronc protein, which could allow enough proteolytically competent Dronc to persist in carrying out functions required for organismal viability. Use of one of the new genetically encoded caspase sensors would allow testing of this possibility [27].
